# MIGRATION ACROSS THE LIFE COURSE AMONG OLDER ADULTS FROM PUERTO RICO

**DOI:** 10.1093/geroni/igac059.048

**Published:** 2022-12-20

**Authors:** Maricruz Rivera-Hernandez

**Affiliations:** Brown University School of Public Health, Worcester, Massachusetts, United States

## Abstract

The prior literature regarding migration among Hispanics to US states has largely focused on younger migrants and often excluded people from Puerto Rico (e.g., Hispanic Paradox; Bostean, 2013). However, older Puerto Ricans appear to be moving to the US mainland (US Census Bureau, 2020, 2021). Therefore, the primary objective of this research was to examine migration among Medicare beneficiaries from Puerto Rico. Using data from Medicare, our results showed that migration trends among Medicare beneficiaries from Puerto Rico have increased over the past decade, including those with special needs. There were 5,593 (1%) Medicare beneficiaries who migrated in 2009 compared to 12,382 (~2%) beneficiaries who migrated in 2018. Among those who migrated, 3,679 were 65 and older in 2009 compared to 8,805 in 2018. The majority of older adults who moved to US states were female (59%), about 75 years old and were enrolled in Medicare Advantage. Destination states among Medicare beneficiaries from Puerto Rico included Florida, New York and New Jersey (comparable to those reported for the general population of Puerto Rico in the US Census). This research has policy implications. Although people from Puerto Rico have different reasons for migrating, those with long term care needs may have multiple varying reasons to move to the mainland. However, migration to the US may have additional challenges for people with high needs (Mellgard et al., 2019). For instance, Medicare coverage/network may not transfer to the US and plan switching/disenrolling may not be a straightforward process for people leaving Puerto Rico.

